# Non-Viable *Lactobacillus reuteri* DSMZ 17648 (Pylopass™) as a New Approach to *Helicobacter pylori* Control in Humans

**DOI:** 10.3390/nu5083062

**Published:** 2013-08-02

**Authors:** Heidrun Mehling, Andreas Busjahn

**Affiliations:** 1 Experimental and Clinical Research Center, Charité Campus Berlin-Buch (CCB), Lindenberger Weg 80, Berlin 13125, Germany; E-Mail: heidrun.mehling@charite.de; 2 HealthTwiSt GmbH, Lindenberger Weg 80, Berlin 13125, Germany

**Keywords:** *Helicobacter pylori*, *Lactobacillus reuteri*, urea breath test

## Abstract

Prevalence of infections by *Helicobacter pylori*, a pathogen involved in a number of gastrointestinal diseases, remains high in developing countries. Management of infections by eradication is not always an option. *Lactobacillus reuteri* (*L. reuteri*) DSMZ17648 (Pylopass™/Lonza) specifically co-aggregates *H. pylori* *in vitro* and was shown to reduce ^13^C urea breath test *in vivo*. In this pilot study, we tried to replicate previous findings in an independent sample and to evaluate effects of spray-drying *vs.* freeze-drying of cultures. A single-blinded, placebo-controlled study was done in 22 *H. pylori* positive, asymptomatic adults. *H. pylori* levels were determined by ^13^C-urea-breath method after 14 days of supplementation, as well as after 6, 12, and 24 weeks follow-up. In the test group, but not in the placebo group, a significant reduction of *H. pylori* was observed. For the first time, spray-dried cells of *L. reuteri* DSMZ17648 have been used in a human study and results are in line with the first study results, supplementing with freeze-dried material. This is of special interest as spray-drying results in dead cell material, meaning that the effect of *L. reuteri* must be independent of its probiotic activity. These results confirm the potential of Pylopass™ as a novel way to reduce the load of *H. pylori*.

## 1. Introduction

*H. pylori* is a gram negative, spiral-shaped human pathogen infecting an estimated 50% of the global population. There is great disparity in the prevalence of infection between developed and developing nations. The average prevalence in developed countries in those <40 years old is 20% whereas developing countries have a prevalence rate of 80%–90% [1]. This makes measures for control most relevant for regions, such as South and Far East Asia, Africa and Latin America.

*H. pylori* is able to survive the acidic environment of the stomach and to adhere to the gastric mucosa, colonizing the mucosal lining of the stomach. An estimated 10^4^–10^7^ *H. pylori* colony forming units (CFU) per g of gastric mucus can be found in infected persons [2].

*H. pylori* is associated with a number of gastrointestinal diseases, such as peptic ulcer disease and gastric cancer [3]. Infection by *H. pylori* may lead to an inflammatory response, increased secretion of gastric acid, and type-B gastritis. There is evidence of a relationship between the level of gastric colonization by *H. pylori* and the probability of symptoms/onset of disease [4]. In the National Health and Nutrition Examination Survey III, *H pylori* was not associated with all-cause mortality. *H pylori* was strongly positively related to gastric cancer mortality. There was an inverse association of *H pylori* status with stroke mortality, pointing to possible protective effects [5].

The management of *H. pylori* infections is still a matter of discussion. The fourth edition of the Maastricht Consensus Report provides diagnostic guidelines and therapeutic strategies for *H. pylori* infection [6]. In dyspeptic patients, a test-and-treat strategy is proposed. Therapeutic options for *H. pylori*-infection aiming at complete eradication include various combinations of proton pump inhibitors combined with two to three antibiotics. This complex approach has inherently high risks of side effects and non-compliance. Furthermore, this push towards eradication has been challenged for developing countries like India where high prevalence, increasing resistance, diversity of strains, together with high risk of recurring infections would make test-and-treat potentially more harmful than beneficial at the community level [7]. The Maastricht Consensus Report also does not state that eradication treatment is indicated for asymptomatic individuals with *H. pylori*.

Therefore, there remains a therapeutic gap for persons infected by *H. pylori*, but still without clinically relevant pathology [8].

The efficacy of using probiotics in the prevention and treatment of various gastrointestinal diseases has been summarized in a recent review, confirming the potential for beneficial health effects [9]. These benefits could apply to the treatment of infections by *H. pylori* as well. Of special interest in that context is the increasing evidence that not just living probiotics but also dead cells or even cell fractions seem to be sufficient to modify biological responses. Adams reports several cases of heat killed probiotic strains exerting positive influences, including reduction of cholesterol, attenuation of allergic response and pain modulation. [10].

The aim of the development of a specific *Lactobacillus* is to close the therapeutic gap in asymptomatic infection and to provide new preventive options to patients by lowering the risk for gastric ulcer or carcinoma, thereby avoiding severe adverse effects and treatment expenses. In a previous placebo-controlled proof-of-concept *in vivo* study we tested *Lactobacillus reuteri* strain DSMZ17648 (Pylopass™/Lonza) [11]. This specific strain was found by screening hundreds of *Lactobacilli* strains of a large culture collection (Organobalance GmbH, Berlin, Germany). This strain was tested for antibiotic resistance and no resistance was identified. A significant reduction in ^13^C-urea breath test (UBT) indicated reduction of bacteria load after active treatment with freeze-dried cells, while no effects were found for placebo control in asymptomatic humans after the two-week supplementation period. *Lactobacillus reuteri* is found in both human breast milk as well as the microflora of the gastrointestinal tract. Strains of *L. reuteri* have been shown to confer health benefits in a variety of cases, including infant colic, gastrointestinal disorders in children and feeding intolerance in pre-term infants [12]. Inhibitory effects of *L. reuteri* (ATCC 55730) on *H. pylori* have been reported as well [13].

The primary objective of this study was to replicate in an independent sample previous findings of the impact of two weeks of Pylopass™ supplementation on *H. pylori* load as measured by ^13^C-UBT. The secondary objective of this study was to evaluate whether a change in the manufacturing process of *L. reuteri* DSMZ17648 from freeze-drying to spray-drying impacts the effectiveness of the supplement. Spray-drying does have obvious advantages over freeze-drying not only with regard to production cost but also with regard to storage stability of the product.

## 2. Experimental Section

The study was approved by the local ethics advisory committee (Charité, Berlin, Germany). The study was conducted according to the Declaration of Helsinki and is registered at ISRCTN (International Standard Randomised Controlled Trial Number ISRCTN70607306).

### 2.1. Study Population

Sample size was estimated in a power calculation as 20 subjects; allowing for potential drop-outs this number was increased by 10%. The study population included 22 *H. pylori* positive subjects (5 male, 17 female, mean age 47 ± 16). Subjects were included if they had reached the age of 18, had documented informed consent, a positive finding in the antibody-based screening test (Diagnostik Nord, Schwerin, Germany), and a positive *H. pylori* finding in the ^13^C urea breath test (Helicobacter Test INFAI^®^, (INFAI GmbH, Köln, Germany), δ ≥ 12‰). Exclusion criteria were intake of any medication interfering with the action of the lactobacilli, previous surgical procedures affecting stomach or small intestine with potential interference with the study, e.g., gastrectomy or gastric bypass, diabetes type 1 or 2, familiar lipid metabolism diseases, liver disease, kidney insufficiency, autoimmune disease, organ transplantation, weight changes >3 kg over the last three months, eradication therapy, lactose intolerance, oral intake of antibiotics <3 months ago, intake of PPIs or H2 antagonists, pregnancy or lactation, alcohol or drug abuse, psychiatric diseases or participation at other clinical trials at the same time. None of the participants met the criteria set forth by the Maastricht Guidelines for eradication therapy [6].

### 2.2. Study Supplements

The test product (active ingredient) consisted of spray-dried dead cells of the *Lactobacillus reuteri* strain DSMZ17648, prepared as solid tablets (Quimifarma S.L., Yuncos (Toledo), Spain) for oral application. Each tablet contained 5 × 10^9^ cells and the daily dosage of 4 tablets translates into 2 × 10^10^ cells. Supplement and placebo tablets were identical in weight (250 mg), size, color and flavor.

### 2.3. Study Protocol

Supplement and placebo were given in a single-blinded, crossover design. All tests were done at the clinical research center of the Charité in Berlin-Buch. Following a first urea breath test, there were 14 days of placebo-run in. After a second breath test, the supplement was given for 14 days, followed by a third breath test. To test mid-term effects, follow-up measures were taken at 6, 12, and 24 weeks after the end of supplementation.

Subjects were instructed to take two tablets after breakfast as well as after their evening meal. During the supplementation period participants were instructed not to initiate any lifestyle or dietary changes.

Subjects were asked to answer a study-specific questionnaire to document well-being, any potential side effects, smoking, alcohol use, nutrition and medication.

To test for physiological changes relevant in terms of safety, fasting blood samples were taken before and after the supplementation period to determine levels of cholesterol, triglycerides, glucose, gamma-GT, GPT (ALAT), GOT (ASAT), creatinine, erythrocytes, hematocrit, hemoglobin, MCV (mean corpuscular volume), MCHC (mean corpuscular hemoglobin concentration), leukocytes, thrombocytes, total bilirubin, uric acid, urea, CRP (C-reactive protein), and the coagulation parameters INR (international normalized ratio) and PTT (partial thromboplastin time).

### 2.4. Outcome Measurements

Detection of *H. pylori* infection in the screening phase was performed with an antibody-based quick test (Diagnostik Nord, Schwerin, Germany). Quantification of colonization for confirmation of eligibility and to verify effects of DSMZ17648 was accomplished by a breath test, as this diagnostic approach is best suited to detection of intraindividual changes [14]. Helicobacter Test INFAI^®^ (INFAI GmbH, Köln, Germany) is a breath test for direct non-invasive semi-quantitative detection of the bacterium *H. pylori* [15]. The test is based on the urease activity of *H. pylori*. Patients ingest 75 mg of the ^13^C urea isotope. In the presence of *H. pylori*, ^13^C urea is hydrolyzed to ammonium and ^13^C-labelled carbon dioxide, which is detectable by mass spectroscopy in the breath. As there is a small amount of naturally occurring ^13^C present in the exhalation air even in the absence of urease activity, breath samples are taken before and 30 min after the ingestion of ^13^C urea. An infection with *H. pylori* is regarded as proven if the difference in ^13^C/^12^C of 0-min-value and 30-min-value exceeds 4‰. If there is no difference, the test is negative, indicating no infection with *H. pylori*. There is a quantitative relation between urease activity and amount of ^13^C in breath in fasted subjects, that indirectly relates to the level of colonization by *H. pylori*, making it well suited to detect reduction or eradication of the bacteria [15,16]. In this study, subjects were included if their breath test value was >12‰, indicating a moderately high colonization level of *H. pylori*. Specificity (98.5%) and sensitivity (97.9%) of Helicobacter Test INFAI^®^ are comparable to traditional invasive diagnostic methods (endoscopy or biopsy) [17].

### 2.5. Statistics

All data were entered into a dedicated trial database. Statistical analysis was conducted using R (Version 3.0.1, R Foundation for Statistical Computing, Vienna, Austria) [18]. We computed differences in ^13^C Urea Breath Test (UBT) values against initial measurements: ∆active = ^13^C UBT active − ^13^C UBT initial, ∆placebo = ^13^C UBT placebo − ^13^C UBT initial. Additionally, the absolute test values between the various study time-points were compared: ^13^C UBT initial, ^13^C UBT active (after 14 days active treatment), ^13^C UBT placebo (after 14 days placebo treatment).

Due to the selection criteria (^13^C UBT > 12‰ at baseline), deviation from normal distribution was assumed and differences were tested by Skillings-Mack test for multiple repeated measurements. If significant, pairwise Wilcoxon tests were applied to distinguish placebo and treatment effects. An error level of 5% was set as threshold for significance. Results are reported as median and 1st/3rd quartile.

## 3. Results

### 3.1. Reduction of *H. pylori*

Screening included 364 subjects, of which 22 subjects had a positive quick test and a ^13^C UBT result above the threshold of 12‰, indicating at least moderately high colonization by *H. pylori*. Details of the screening population are given in Table 1.

**Table 1:** Characteristics of screening population.

	Total	Included	Excluded
*n*	364	22	342
Age (years ± SD)	47 ± 20	47 ± 16	47 ± 20
Sex (male/female)	108/256	5/17	103/239

**Figure 1:**
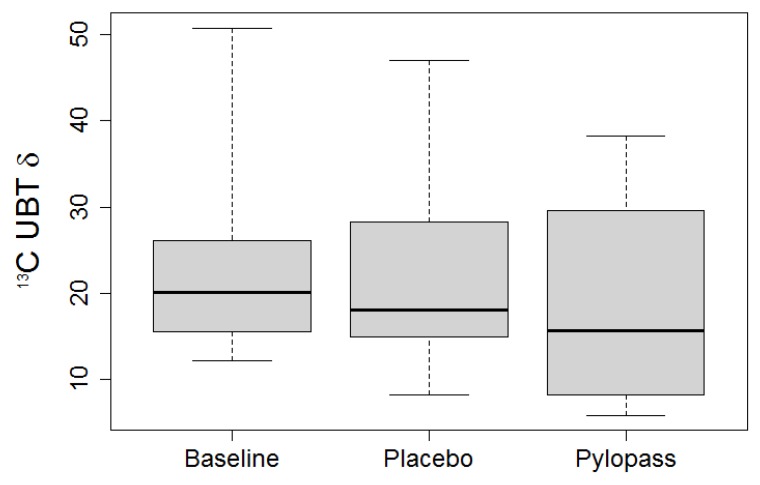
Absolute ^13^C-urea breath test (UBT) values before and after supplementation with placebo or Pylopass™.

Overall, 22 subjects started supplementation, with no drop-outs during the trial phase. There was large interindividual variability of quantitative measures of colonization at baseline as well as during treatment, together with substantial intraindividual variability between measurements. Main analysis of *H. pylori* reduction by DSMZ17648 was based on median values of absolute values of ^13^C UBT at baseline measurement, after placebo and after active treatment, Figure 1.

Results for the ^13^C UBT are reported in Table 2, including absolute values during trial phases as well as absolute and relative differences between baseline and supplementation phases. Only the difference between ^13^C UBT at baseline (median = 20) and after DSMZ17648 supplementation (median = 16) reached statistical significance (*p* = 0.011), while baseline and placebo (median = 18) did not differ (*p* = 0.237). There was a statistical trend for DSMZ17648 values to be lower than placebo (*p* = 0.084).

**Figure 2:**
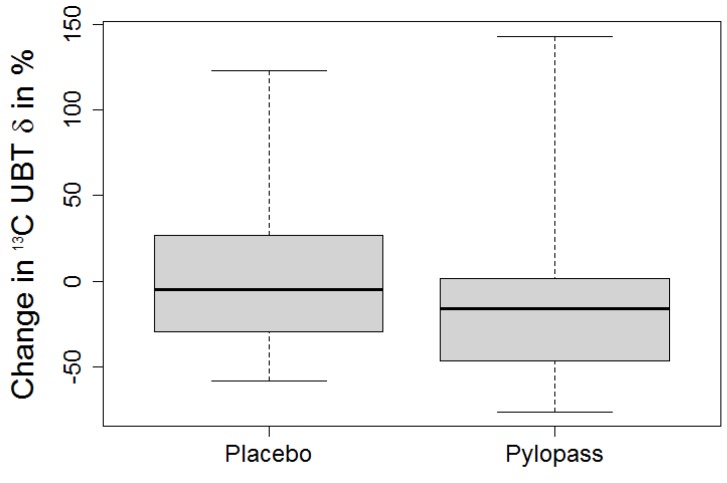
Percentage of change of 13C UBT after placebo and Pylopass™.

We calculated individual absolute differences as well as change scores (change in % of baseline). For DSMZ17648, both absolute difference and change score are significant (*p* = 0.018 and 0.013, respectively), indicating a reduction of *H. pylori* (Figure 2). For placebo, neither absolute nor relative changes are significantly differing from 0, suggesting random variation rather than a systematic reduction.

**Table 2:** Breath test findings in study population, results are reported as median (25./75. percentile).

	Baseline	Placebo	Pylopass™
Absolute values (‰)	20 (16/26)	18 (15/28)	16 (8/30) *^,+^
Absolute change *vs.* baseline (‰)		0 (−6/3)	−5 (−11/0) ^+^
Relative change *vs.* baseline (%)		−3 (−27/20)	−16 (−47/1) ^+^

* *p* < 0.05 *vs.* baseline; ^+^ *p* < 0.1 *vs.* placebo.

**Figure 3:**
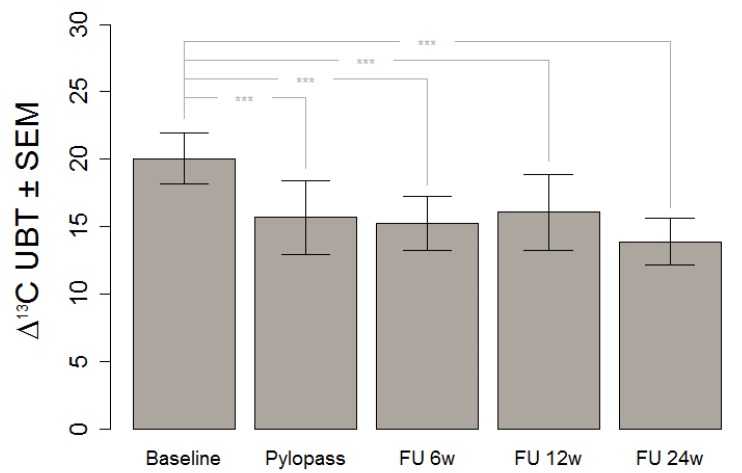
Absolute ^13^C UBT values before and after supplementation with Pylopass™ as well during follow-up.

We tested prolonged effects by repeating the ^13^C UBT at 6, 12, and 24 weeks after the end of supplementation. Results are shown in Figure 3, the significant lowering found immediately after supplementation was still detectable after 24 weeks.

After supplementation with *L. reuteri* DSMZ17648, not all subjects showed a reduction in *H. pylori* colonization. Responses show some variability, studying potential predictors for response may define a subgroup of patients most likely to benefit. This will require substantially larger study groups in the future.

### 3.2. Safety Parameters

No side effects were reported during 14 days of either placebo or DSMZ17648 supplementation. There were no statistically significant alterations in a complete blood cell profile or in circulating metabolic enzymes with markers of liver and renal function measured as safety parameters before and after the supplementation period. During the course of the study no changes in lifestyle (physical activity, diet), or in general health were detected as indicated by the questionnaire.

### 3.3. Effects of Spray-Drying

**Figure 4:**
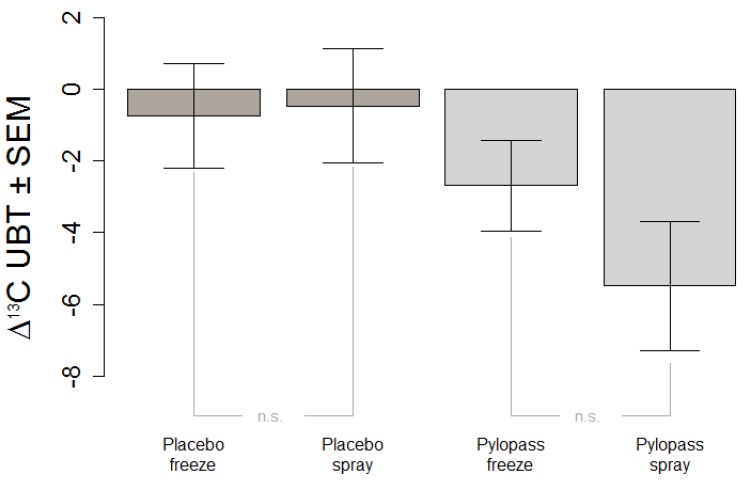
Comparison of freeze-dried and spray-dried *L. reuteri* supplement in terms of change of 13C UBT after placebo and Pylopass™.

A secondary objective of this study was to evaluate a change in the preparation of the test culture, *i.e.*, a shift from freeze-drying cells to spray-drying. We compared the absolute change in ^13^C UBT between a previous study with freeze-dried DSMZ17648 [11] and our current results (Figure 4). The results clearly indicate that the process of spray-drying is equally effective. Both types of preparation of non-viable lactobacillus cells show significant reduction in *H. pylori*.

## 4. Discussion

Despite the causal link between *H. pylori* infections and gastrointestinal pathology, only a minority of infected subjects actually develops disease [19]. For those with acute symptoms, eradication in a test-and treat approach is usually recommended as the standard procedure. Yet on the population level, this may neither be necessary nor achievable, especially in regions with high prevalence and poorly developed health systems. Given the interaction between infections with *H. pylori* and mental stress in gastrointestinal diseases [20], short-term reduction may be another viable option during stressful life events, such as, e.g., exams, excessive work load, or other emotional distress.

This *in vivo* study shows a significant decrease of *H. pylori* stomach colonization after *Lactobacillus* *reuteri* DSMZ17648 supplementation in asymptomatic subjects with detectable *H. pylori* infection. It replicates our previous findings in an independent general population sample free of overt gastrointestinal diseases or alarm symptoms. Primary objective of this study was the reduction of *H. pylori* as measured by ^13^C urease breath test (Helicobacter Test INFAI^®^) after a 14 day supplementation period of *L. reuteri* DSMZ17648 (Pylopass™) at a daily dose of 2 × 10^10^ non-viable spray-dried cells.

This finding gives a strong foundation for the assumption that consumption of DSMZ17648 might exert a preventive effect of secondary diseases and related symptoms due to *H. pylori* infection. It is of special relevance that the preventive effect is carried over well after the supplementation itself, at least for the six month period covered by our follow-up.

Probiotics are defined as live microorganisms which when administered in adequate amounts confer a health benefit to the host (FAO/WHO) [21]. In the context of *H. pylori* infection, probiotics are administered along with eradication therapy to ease side-effects. Medeiros *et al.* [22] studied the impact of *Lactobacillus acidopholus* on side effects associated with a common triple regimen eradication therapy but found no measurable effect. However, there are some studies where probiotics are used alone in the treatment of *H. pylori*, based on a broad range of cultures and outcomes. Francavilla *et al.* supplemented a different strain of *Lactobacillus reuteri* (ATCC 55730) for four weeks and found a significant reduction in *H. pylori* load [13]. As they had included patients showing some gastrointestinal symptoms, they could verify a reduction in symptom scores during treatment. A study applying *L. casei* for three weeks reported a non-significant suppressive effect [23]. Fourteen *H. pylori*-positive subjects receiving drinks with 10^8^ colony-forming units/mL *L. casei* thrice daily during meals for three weeks were compared to six untreated *H. pylori*-positive subjects as controls. Urease activity decreased in nine of the 14 (64%) subjects with *L. casei* supplementation and in two of the six (33%) controls (*p* = 0.22). A slight, but nonsignificant trend towards a suppressive effect of *L. casei* on *H. pylori* *in vivo* may exist. *In vitro* experiments showed that viable *L. casei* are required for *H. pylori* growth inhibition.

Reduction of *H. pylori* with *L. brevis* administration has also been reported [24]. Twenty-two *H. pylori*-positive dyspeptic patients randomly (ratio 1:1) received high oral doses of lyophilisated *L. brevis* (CD2) or placebo nine times a day for three weeks. *L. brevis* (CD2) treatment did not eradicate *H. pylori*. However, a reduction in the UBT delta values occurred, suggesting a decrease in intragastric bacterial load.

In the case of *L. acidophilus* (*johnsonii*) La1 the whey-based supernatant was found to induce a marked decrease in *H. pylori* breath test analyses, both in combination with Omeprazol and alone [25]. While these studies showed some promising results, further research is needed to determine the efficacy of such methodology in a general population.

Potential mechanisms by which probiotics may influence *H. pylori* may include strengthening of the non-immunological barriers representing a first line of defense against pathogenic bacteria by producing antimicrobial substances, or stabilization of the gut mucosal barrier. Other mechanisms could be sequestration of *H. pylori*, competitive binding to adhesion receptors for *H. pylori* [26,27], binding to surface structures of *H. pylori*, thus preventing adhesion to the mucosa. Antimicrobial substances have been implicated in the inhibition of *H. pylori* by lactic acid bacteria. Short chain fatty acids (SCFAs, e.g., formic, acetic, propionic, butyric and lactic acid) have an important role in decreasing the pH, and are produced during the catabolism of carbohydrates. Such antimicrobial activity could either be due to a direct effect on *H. pylori* but also a secondary effect of the inhibition of its urease activity [28]. Current research suggested that living cells may not be mandatory for health effects, as dead cells, cell fractions, or supernatant may be as efficient in some cases. For the specific strain of *L. reuteri* studied in this project, supplementation of both freeze-dried and spray-dried cells has been shown to reduce *H. pylori*. The most plausible explanation is that structures on the cell surface of this selected strain are responsible for the therapeutic effect and were unaffected by the different drying processes.

DSMZ17648 specifically co-aggregates *H. pylori* under *in vitro* conditions and in artificial gastric juice, without interfering with other bacteria of the commensal intestinal flora [29]. This specific binding may mask surface structures of *H. pylori* and interfere with *Helicobacter* motility. The aggregated pathogens presumably no longer adhere to the gastric mucosa and are cleared from the stomach. An additional mode of action may be competition for specific binding proteins [30].

The present study should be regarded as a pilot trial, as sample size was limited. Larger clinical studies should be conducted in the future to validate these results. Establishment of predictors for response to DSMZ17648 as well as more precise estimation of effect size will require larger samples. Although most other human trials [22,23,24,25] have used the 13C urea breath test to study the effects of probiotics on *H. pylori* colonization, the outcome of ^13^C Urea Breath Test reflects *H. pylori* colonization density in a semi-quantitative way only, making intraindividual changes in test scores only a proxy for intervention effects. Deviation from fasting conditions may influence test outcomes as well [31].

## 5. Conclusions

There are many potential applications of *L. reuteri* DSMZ17648. It may reduce *H. pylori* load in high prevalence populations or be used as short-term prophylaxis during high stress periods. *L. reuteri* DSMZ17648 could also be utilized for chronic, long-term prophylaxis.

Providing access to probiotic cultures in the form of supplements and food products requires sophisticated supply chains which may not be available in regions with the highest need. Dead cells, such as the ones used in the present study, are more stable than true probiotics. Therefore, they can be applied in a wider range of delivery systems to populations which may lack proper storage. *L. reuteri* DSMZ17648 may also be useful in populations with high *H. pylori* prevalence where antibiotic therapy has low compliance for cost reasons. Compared to living probiotic cells, dead cells are advantageous in that storage and delivery is less demanding, shelf life is prolonged and production costs are reduced. Taken together, these characteristics could make the application of dead cells a realistic new approach to *H. pylori* control.
